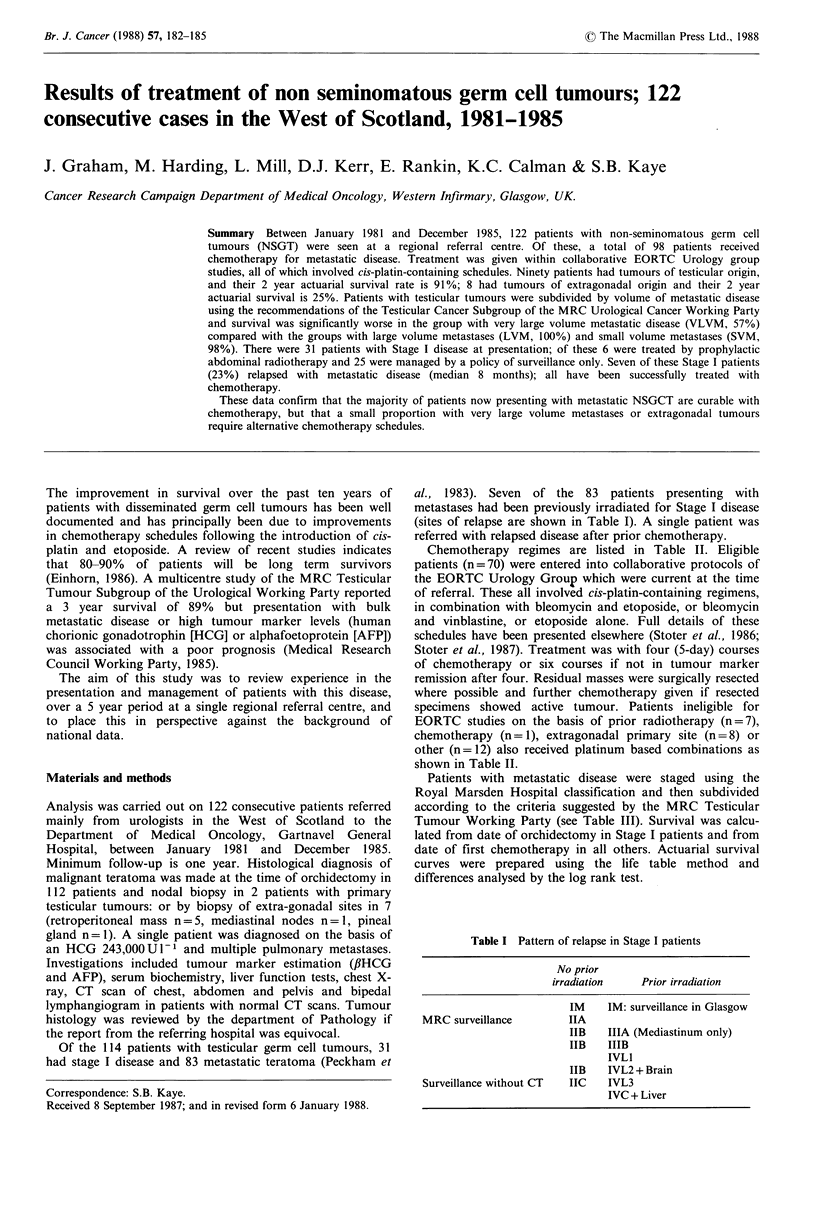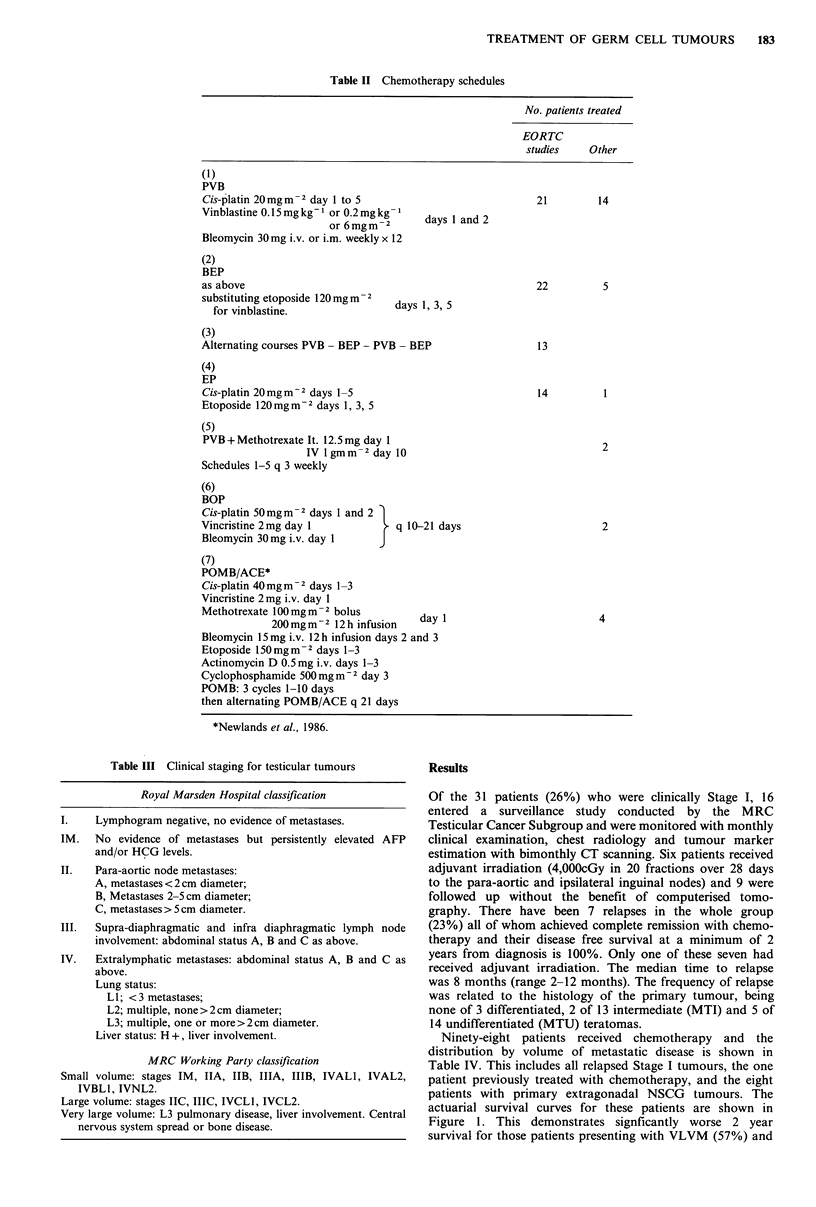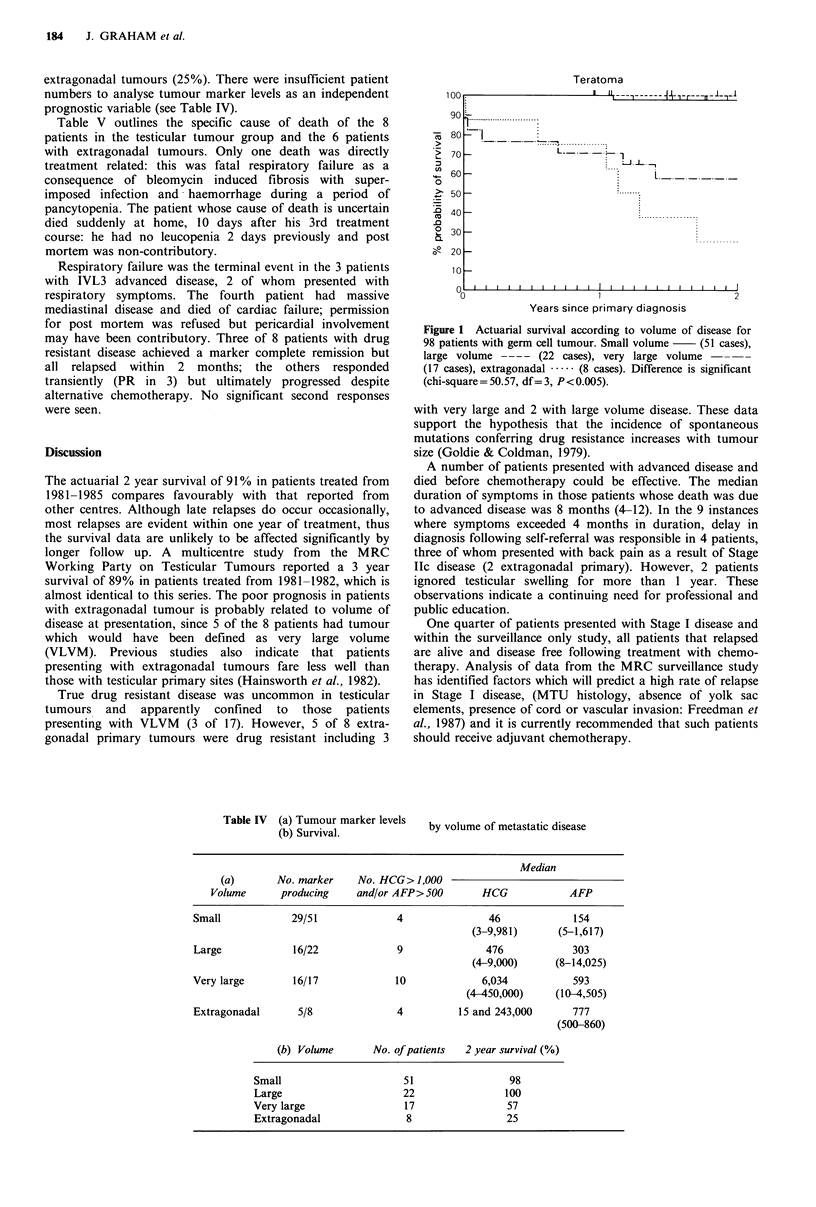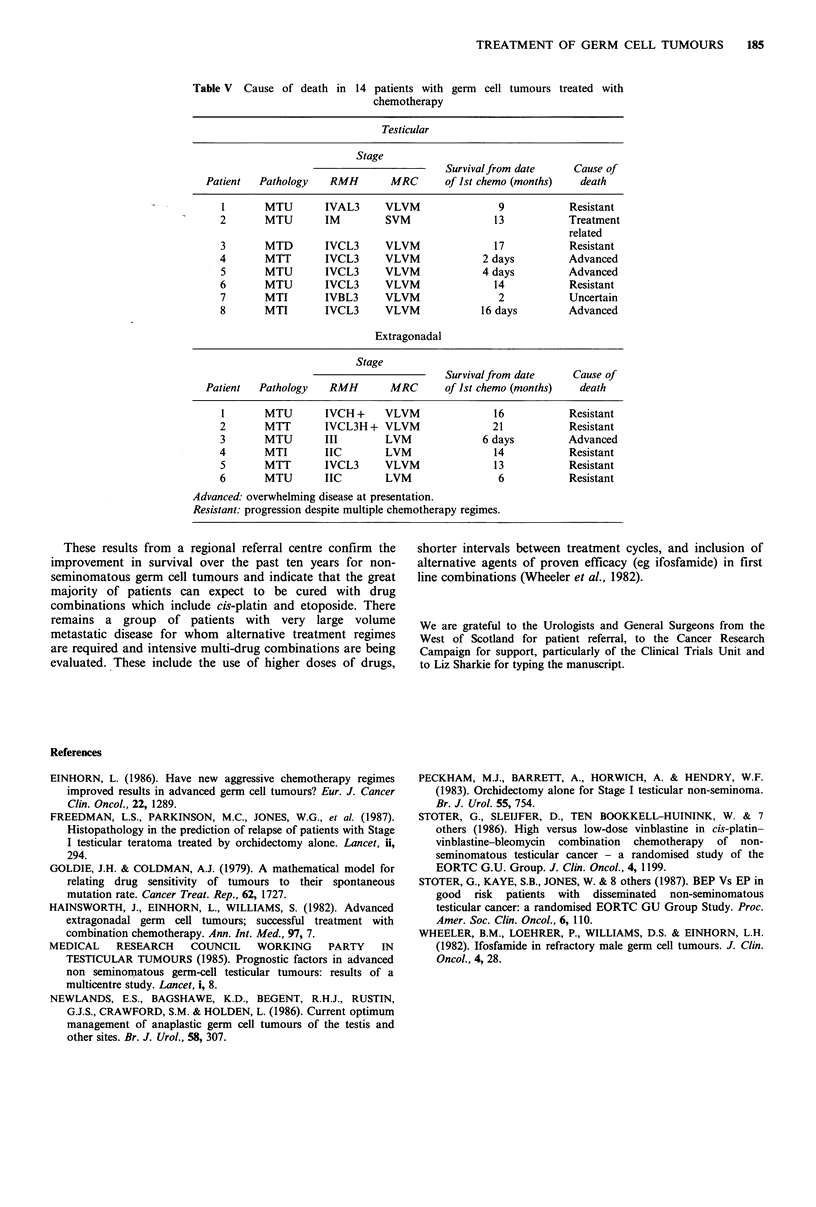# Results of treatment of non seminomatous germ cell tumours; 122 consecutive cases in the West of Scotland, 1981-1985.

**DOI:** 10.1038/bjc.1988.38

**Published:** 1988-02

**Authors:** J. Graham, M. Harding, L. Mill, D. J. Kerr, E. Rankin, K. C. Calman, S. B. Kaye

**Affiliations:** Cancer Research Campaign Department of Medical Oncology, Western Infirmary, Glasgow, UK.

## Abstract

Between January 1981 and December 1985, 122 patients with non-seminomatous germ cell tumours (NSGT) were seen at a regional referral centre. Of these, a total of 98 patients received chemotherapy for metastatic disease. Treatment was given within collaborative EORTC Urology group studies, all of which involved cis-platin-containing schedules. Ninety patients had tumours of testicular origin, and their 2 year actuarial survival rate is 91%; 8 had tumours of extragonadal origin and their 2 year actuarial survival is 25%. Patients with testicular tumours were subdivided by volume of metastatic disease using the recommendations of the Testicular Cancer Subgroup of the MRC Urological Cancer Working Party and survival was significantly worse in the group with very large volume metastatic disease (VLVM, 57%) compared with the groups with large volume metastases (LVM, 100%) and small volume metastases (SVM, 98%). There were 31 patients with Stage I disease at presentation; of these 6 were treated by prophylactic abdominal radiotherapy and 25 were managed by a policy of surveillance only. Seven of these Stage I patients (23%) relapsed with metastatic disease (median 8 months); all have been successfully treated with chemotherapy. These data confirm that the majority of patients now presenting with metastatic NSGCT are curable with chemotherapy, but that a small proportion with very large volume metastases or extragonadal tumours require alternative chemotherapy schedules.


Br. J. Cancer (1988) 57, 182–185 © The Macmillan Press Ltd., 1988

### Results of treatment of non seminomatous germ cell tumours; 122 consecutive cases in the West of Scotland, 1981–1985

J. Graham, M. Harding, L. Mill, D.J. Kerr, E. Rankin, K.C. Calman & S.B. Kaye

*Cancer Research Campaign Department of Medical Oncology, Western Infirmary, Glasgow, UK.*

**Summary** Between January 1981 and December 1985, 122 patients with non-seminomatous germ cell tumours (NSGT) were seen at a regional referral centre. Of these, a total of 98 patients received chemotherapy for metastatic disease. Treatment was given within collaborative EORTC Urology group studies, all of which involved *cis*-platin-containing schedules. Ninety patients had tumours of testicular origin, and their 2 year actuarial survival rate is 91%; 8 had tumours of extragonadal origin and their 2 year actuarial survival is 25%. Patients with testicular tumours were subdivided by volume of metastatic disease using the recommendations of the Testicular Cancer Subgroup of the MRC Urological Cancer Working Party and survival was significantly worse in the group with very large volume metastatic disease (VLVM, 57%) compared with the groups with large volume metastases (LVM, 100%) and small volume metastases (SVM, 98%). There were 31 patients with Stage I disease at presentation; of these 6 were treated by prophylactic abdominal radiotherapy and 25 were managed by a policy of surveillance only. Seven of these Stage I patients (23%) relapsed with metastatic disease (median 8 months); all have been successfully treated with chemotherapy.

These data confirm that the majority of patients now presenting with metastatic NSGCT are curable with chemotherapy, but that a small proportion with very large volume metastases or extragonadal tumours require alternative chemotherapy schedules.

The improvement in survival over the past ten years of patients with disseminated germ cell tumours has been well documented and has principally been due to improvements in chemotherapy schedules following the introduction of *cis*-platin and etoposide. A review of recent studies indicates that 80–90% of patients will be long term survivors (Einhorn, 1986). A multicentre study of the MRC Testicular Tumour Subgroup of the Urological Working Party reported a 3 year survival of 89% but presentation with bulk metastatic disease or high tumour marker levels (human chorionic gonadotrophin [HCG] or alphafoetoprotein [AFP]) was associated with a poor prognosis (Medical Research Council Working Party, 1985).

The aim of this study was to review experience in the presentation and management of patients with this disease, over a 5 year period at a single regional referral centre, and to place this in perspective against the background of national data.

### Materials and methods

Analysis was carried out on 122 consecutive patients referred mainly from urologists in the West of Scotland to the Department of Medical Oncology, Gartnavel General Hospital, between January 1981 and December 1985. Minimum follow-up is one year. Histological diagnosis of malignant teratoma was made at the time of orchidectomy in 112 patients and nodal biopsy in 2 patients with primary testicular tumours: or by biopsy of extra-gonadal sites in 7 (retroperitoneal mass n = 5, mediastinal nodes n = 1, pineal gland n = 1). A single patient was diagnosed on the basis of an HCG 243,000 $U l^{-1}$ and multiple pulmonary metastases. Investigations included tumour marker estimation ($\beta$HCG and AFP), serum biochemistry, liver function tests, chest X-ray, CT scan of chest, abdomen and pelvis and bipedal lymphangiogram in patients with normal CT scans. Tumour histology was reviewed by the department of Pathology if the report from the referring hospital was equivocal.

Of the 114 patients with testicular germ cell tumours, 31 had stage I disease and 83 metastatic teratoma (Peckham *et al.*, 1983). Seven of the 83 patients presenting with metastases had been previously irradiated for Stage I disease (sites of relapse are shown in Table I). A single patient was referred with relapsed disease after prior chemotherapy.

Chemotherapy regimes are listed in Table II. Eligible patients (n = 70) were entered into collaborative protocols of the EORTC Urology Group which were current at the time of referral. These all involved *cis*-platin-containing regimens, in combination with bleomycin and etoposide, or bleomycin and vinblastine, or etoposide alone. Full details of these schedules have been presented elsewhere (Stoter *et al.*, 1986; Stoter *et al.*, 1987). Treatment was with four (5-day) courses of chemotherapy or six courses if not in tumour marker remission after four. Residual masses were surgically resected where possible and further chemotherapy given if resected specimens showed active tumour. Patients ineligible for EORTC studies on the basis of prior radiotherapy (n = 7), chemotherapy (n = 1), extragonadal primary site (n = 8) or other (n = 12) also received platinum based combinations as shown in Table II.

Patients with metastatic disease were staged using the Royal Marsden Hospital classification and then subdivided according to the criteria suggested by the MRC Testicular Tumour Working Party (see Table III). Survival was calculated from date of orchidectomy in Stage I patients and from date of first chemotherapy in all others. Actuarial survival curves were prepared using the life table method and differences analysed by the log rank test.

**Table I** Pattern of relapse in Stage I patients

| | *No prior irradiation* | *Prior irradiation* |
|---|---|---|
| | IM | IM: surveillance in Glasgow |
| MRC surveillance | IIA | |
| | IIB | IIIA (Mediastinum only) |
| | IIB | IIIB |
| | | IVL1 |
| | IIB | IVL2 + Brain |
| Surveillance without CT | IIC | IVL3 |
| | | IVC + Liver |

Correspondence: S.B. Kaye.
Received 8 September 1987; and in revised form 6 January 1988.

**Table II** Chemotherapy schedules

| | | No. patients treated | |
|---|---|---|---|
| | | EORTC studies | Other |
| (1) PVB<br>*Cis*-platin 20 mg m$^{-2}$ day 1 to 5 | | 21 | 14 |
| Vinblastine 0.15 mg kg$^{-1}$ or 0.2 mg kg$^{-1}$ or 6 mg m$^{-2}$ | days 1 and 2 | | |
| Bleomycin 30 mg i.v. or i.m. weekly x 12 | | | |
| (2) BEP<br>as above | | 22 | 5 |
| substituting etoposide 120 mg m$^{-2}$ for vinblastine. | days 1, 3, 5 | | |
| (3) Alternating courses PVB - BEP - PVB - BEP | | 13 | |
| (4) EP<br>*Cis*-platin 20 mg m$^{-2}$ days 1–5 | | 14 | 1 |
| Etoposide 120 mg m$^{-2}$ days 1, 3, 5 | | | |
| (5) PVB + Methotrexate It. 12.5 mg day 1<br>IV 1 gm m$^{-2}$ day 10 | | | 2 |
| Schedules 1–5 q 3 weekly | | | |
| (6) BOP<br>*Cis*-platin 50 mg m$^{-2}$ days 1 and 2<br>Vincristine 2 mg day 1<br>Bleomycin 30 mg i.v. day 1 | q 10–21 days | | 2 |
| (7) POMB/ACE*<br>*Cis*-platin 40 mg m$^{-2}$ days 1–3<br>Vincristine 2 mg i.v. day 1 | | | |
| Methotrexate 100 mg m$^{-2}$ bolus<br>200 mg m$^{-2}$ 12 h infusion | day 1 | | 4 |
| Bleomycin 15 mg i.v. 12 h infusion days 2 and 3<br>Etoposide 150 mg m$^{-2}$ days 1–3<br>Actinomycin D 0.5 mg i.v. days 1–3<br>Cyclophosphamide 500 mg m$^{-2}$ day 3<br>POMB: 3 cycles 1–10 days<br>then alternating POMB/ACE q 21 days | | | |

*Newlands *et al.*, 1986.

**Table III** Clinical staging for testicular tumours

*Royal Marsden Hospital classification*

I. Lymphogram negative, no evidence of metastases.

IM. No evidence of metastases but persistently elevated AFP and/or HCG levels.

II. Para-aortic node metastases:
A, metastases < 2 cm diameter;
B, Metastases 2–5 cm diameter;
C, metastases > 5 cm diameter.

III. Supra-diaphragmatic and infra diaphragmatic lymph node involvement: abdominal status A, B and C as above.

IV. Extralymphatic metastases: abdominal status A, B and C as above.
Lung status:
L1; < 3 metastases;
L2; multiple, none > 2 cm diameter;
L3; multiple, one or more > 2 cm diameter.
Liver status: H+, liver involvement.

*MRC Working Party classification*

Small volume: stages IM, IIA, IIB, IIIA, IIIB, IVAL1, IVAL2, IVBL1, IVNL2.

Large volume: stages IIC, IIIC, IVCL1, IVCL2.

Very large volume: L3 pulmonary disease, liver involvement. Central nervous system spread or bone disease.

### Results

Of the 31 patients (26%) who were clinically Stage I, 16 entered a surveillance study conducted by the MRC Testicular Cancer Subgroup and were monitored with monthly clinical examination, chest radiology and tumour marker estimation with bimonthly CT scanning. Six patients received adjuvant irradiation (4,000cGy in 20 fractions over 28 days to the para-aortic and ipsilateral inguinal nodes) and 9 were followed up without the benefit of computerised tomography. There have been 7 relapses in the whole group (23%) all of whom achieved complete remission with chemotherapy and their disease free survival at a minimum of 2 years from diagnosis is 100%. Only one of these seven had received adjuvant irradiation. The median time to relapse was 8 months (range 2–12 months). The frequency of relapse was related to the histology of the primary tumour, being none of 3 differentiated, 2 of 13 intermediate (MTI) and 5 of 14 undifferentiated (MTU) teratomas.

Ninety-eight patients received chemotherapy and the distribution by volume of metastatic disease is shown in Table IV. This includes all relapsed Stage I tumours, the one patient previously treated with chemotherapy, and the eight patients with primary extragonadal NSCG tumours. The actuarial survival curves for these patients are shown in Figure 1. This demonstrates signficantly worse 2 year survival for those patients presenting with VLVM (57%) and extragonadal tumours (25%). There were insufficient patient numbers to analyse tumour marker levels as an independent prognostic variable (see Table IV).

Table V outlines the specific cause of death of the 8 patients in the testicular tumour group and the 6 patients with extragonadal tumours. Only one death was directly treatment related: this was fatal respiratory failure as a consequence of bleomycin induced fibrosis with superimposed infection and haemorrhage during a period of pancytopenia. The patient whose cause of death is uncertain died suddenly at home, 10 days after his 3rd treatment course: he had no leucopenia 2 days previously and post mortem was non-contributory.

Respiratory failure was the terminal event in the 3 patients with IVL3 advanced disease, 2 of whom presented with respiratory symptoms. The fourth patient had massive mediastinal disease and died of cardiac failure; permission for post mortem was refused but pericardial involvement may have been contributory. Three of 8 patients with drug resistant disease achieved a marker complete remission but all relapsed within 2 months; the others responded transiently (PR in 3) but ultimately progressed despite alternative chemotherapy. No significant second responses were seen.

### Discussion

The actuarial 2 year survival of 91% in patients treated from 1981–1985 compares favourably with that reported from other centres. Although late relapses do occur occasionally, most relapses are evident within one year of treatment, thus the survival data are unlikely to be affected significantly by longer follow up. A multicentre study from the MRC Working Party on Testicular Tumours reported a 3 year survival of 89% in patients treated from 1981–1982, which is almost identical to this series. The poor prognosis in patients with extragonadal tumour is probably related to volume of disease at presentation, since 5 of the 8 patients had tumour which would have been defined as very large volume (VLVM). Previous studies also indicate that patients presenting with extragonadal tumours fare less well than those with testicular primary sites (Hainsworth *et al.*, 1982).

True drug resistant disease was uncommon in testicular tumours and apparently confined to those patients presenting with VLVM (3 of 17). However, 5 of 8 extragonadal primary tumours were drug resistant including 3

**Figure 1** Actuarial survival according to volume of disease for 98 patients with germ cell tumour. Small volume —— (51 cases), large volume ---- (22 cases), very large volume —— (17 cases), extragonadal ····· (8 cases). Difference is significant (chi-square = 50.57, df = 3, $P < 0.005$).

with very large and 2 with large volume disease. These data support the hypothesis that the incidence of spontaneous mutations conferring drug resistance increases with tumour size (Goldie & Coldman, 1979).

A number of patients presented with advanced disease and died before chemotherapy could be effective. The median duration of symptoms in those patients whose death was due to advanced disease was 8 months (4–12). In the 9 instances where symptoms exceeded 4 months in duration, delay in diagnosis following self-referral was responsible in 4 patients, three of whom presented with back pain as a result of Stage IIc disease (2 extragonadal primary). However, 2 patients ignored testicular swelling for more than 1 year. These observations indicate a continuing need for professional and public education.

One quarter of patients presented with Stage I disease and within the surveillance only study, all patients that relapsed are alive and disease free following treatment with chemotherapy. Analysis of data from the MRC surveillance study has identified factors which will predict a high rate of relapse in Stage I disease, (MTU histology, absence of yolk sac elements, presence of cord or vascular invasion: Freedman *et al.*, 1987) and it is currently recommended that such patients should receive adjuvant chemotherapy.

**Table IV** (a) Tumour marker levels (b) Survival. by volume of metastatic disease

| (*a*) Volume | *No. marker producing* | *No. HCG > 1,000 and/or AFP > 500* | *Median* HCG | *Median* AFP |
|---|---|---|---|---|
| Small | 29/51 | 4 | 46 (3–9,981) | 154 (5–1,617) |
| Large | 16/22 | 9 | 476 (4–9,000) | 303 (8–14,025) |
| Very large | 16/17 | 10 | 6,034 (4–450,000) | 593 (10–4,505) |
| Extragonadal | 5/8 | 4 | 15 and 243,000 | 777 (500–860) |

| (*b*) *Volume* | *No. of patients* | *2 year survival* (%) |
|---|---|---|
| Small | 51 | 98 |
| Large | 22 | 100 |
| Very large | 17 | 57 |
| Extragonadal | 8 | 25 |

**Table V** Cause of death in 14 patients with germ cell tumours treated with chemotherapy

*Testicular*

| *Patient* | *Pathology* | *Stage* *RMH* | *Stage* *MRC* | *Survival from date of 1st chemo (months)* | *Cause of death* |
|---|---|---|---|---|---|
| 1 | MTU | IVAL3 | VLVM | 9 | Resistant |
| 2 | MTU | IM | SVM | 13 | Treatment related |
| 3 | MTD | IVCL3 | VLVM | 17 | Resistant |
| 4 | MTT | IVCL3 | VLVM | 2 days | Advanced |
| 5 | MTU | IVCL3 | VLVM | 4 days | Advanced |
| 6 | MTU | IVCL3 | VLVM | 14 | Resistant |
| 7 | MTI | IVBL3 | VLVM | 2 | Uncertain |
| 8 | MTI | IVCL3 | VLVM | 16 days | Advanced |

Extragonadal

| *Patient* | *Pathology* | *Stage* *RMH* | *Stage* *MRC* | *Survival from date of 1st chemo (months)* | *Cause of death* |
|---|---|---|---|---|---|
| 1 | MTU | IVCH+ | VLVM | 16 | Resistant |
| 2 | MTT | IVCL3H+ | VLVM | 21 | Resistant |
| 3 | MTU | III | LVM | 6 days | Advanced |
| 4 | MTI | IIC | LVM | 14 | Resistant |
| 5 | MTT | IVCL3 | VLVM | 13 | Resistant |
| 6 | MTU | IIC | LVM | 6 | Resistant |

*Advanced:* overwhelming disease at presentation.
*Resistant:* progression despite multiple chemotherapy regimes.

These results from a regional referral centre confirm the improvement in survival over the past ten years for non-seminomatous germ cell tumours and indicate that the great majority of patients can expect to be cured with drug combinations which include *cis*-platin and etoposide. There remains a group of patients with very large volume metastatic disease for whom alternative treatment regimes are required and intensive multi-drug combinations are being evaluated. These include the use of higher doses of drugs, shorter intervals between treatment cycles, and inclusion of alternative agents of proven efficacy (eg ifosfamide) in first line combinations (Wheeler *et al.*, 1982).

We are grateful to the Urologists and General Surgeons from the West of Scotland for patient referral, to the Cancer Research Campaign for support, particularly of the Clinical Trials Unit and to Liz Sharkie for typing the manuscript.

### References

EINHORN, L. (1986). Have new aggressive chemotherapy regimes improved results in advanced germ cell tumours? *Eur. J. Cancer Clin. Oncol.*, **22**, 1289.

FREEDMAN, L.S., PARKINSON, M.C., JONES, W.G., *et al.* (1987). Histopathology in the prediction of relapse of patients with Stage I testicular teratoma treated by orchidectomy alone. *Lancet*, **ii**, 294.

GOLDIE, J.H. & COLDMAN, A.J. (1979). A mathematical model for relating drug sensitivity of tumours to their spontaneous mutation rate. *Cancer Treat. Rep.*, **62**, 1727.

HAINSWORTH, J., EINHORN, L., WILLIAMS, S. (1982). Advanced extragonadal germ cell tumours; successful treatment with combination chemotherapy. *Ann. Int. Med.*, **97**, 7.

MEDICAL RESEARCH COUNCIL WORKING PARTY IN TESTICULAR TUMOURS (1985). Prognostic factors in advanced non seminomatous germ-cell testicular tumours: results of a multicentre study. *Lancet*, **i**, 8.

NEWLANDS, E.S., BAGSHAWE, K.D., BEGENT, R.H.J., RUSTIN, G.J.S., CRAWFORD, S.M. & HOLDEN, L. (1986). Current optimum management of anaplastic germ cell tumours of the testis and other sites. *Br. J. Urol.*, **58**, 307.

PECKHAM, M.J., BARRETT, A., HORWICH, A. & HENDRY, W.F. (1983). Orchidectomy alone for Stage I testicular non-seminoma. *Br. J. Urol.* **55**, 754.

STOTER, G., SLEIJFER, D., TEN BOOKKELL-HUININK, W. & 7 others (1986). High versus low-dose vinblastine in *cis*-platin–vinblastine–bleomycin combination chemotherapy of non-seminomatous testicular cancer – a randomised study of the EORTC G.U. Group. *J. Clin. Oncol.*, **4**, 1199.

STOTER, G., KAYE, S.B., JONES, W. & 8 others (1987). BEP Vs EP in good risk patients with disseminated non-seminomatous testicular cancer: a randomised EORTC GU Group Study. *Proc. Amer. Soc. Clin. Oncol.*, **6**, 110.

WHEELER, B.M., LOEHRER, P., WILLIAMS, D.S. & EINHORN, L.H. (1982). Ifosfamide in refractory male germ cell tumours. *J. Clin. Oncol.*, **4**, 28.